# Soil Salinity Inversion of Winter Wheat Areas Based on Satellite-Unmanned Aerial Vehicle-Ground Collaborative System in Coastal of the Yellow River Delta

**DOI:** 10.3390/s20226521

**Published:** 2020-11-14

**Authors:** Guanghui Qi, Gengxing Zhao, Xue Xi

**Affiliations:** 1 College of Information Science and Engineering, Shandong Agricultural University, Tai’an 271018, China; qghui@sdau.edu.cn; 2 National Engineering Laboratory for Efficient Utilization of Soil and Fertilizer Resources, College of Resources and Environment, Shandong Agricultural University, Tai’an 271018, China; 2018110265@sdau.edu.cn

**Keywords:** sentinel-2A image, UAV image, remote sensing, soil salinity

## Abstract

Soil salinization is an important factor affecting winter wheat growth in coastal areas. The rapid, accurate and efficient estimation of soil salt content is of great significance for agricultural production. The Kenli area in the Yellow River Delta was taken as the research area. Three machine learning inversion models, namely, BP neural network (BPNN), support vector machine (SVM) and random forest (RF) were constructed using ground-measured data and UAV images, and the optimal model is applied to UAV images to obtain the salinity inversion result, which is used as the true salt value of the Sentinel-2A image to establish BPNN, SVM and RF collaborative inversion models, and apply the optimal model to the study area. The results showed that the RF collaborative inversion model is optimal, R^2^ = 0.885. The inversion results are verified by using the measured soil salt data in the study area, which is significantly better than the directly satellite remote sensing inversion method. This study integrates the advantages of multi-scale data and proposes an effective “Satellite-UAV-Ground” collaborative inversion method for soil salinity, so as to obtain more accurate soil information, and provide more effective technical support for agricultural production.

## 1. Introduction

Soil salinization is a form of soil degradation. Soil salinization will not only cause a series of problems such as ecological deterioration, but also have a negative impact on the growth of crops [1]. Therefore, it is of great practical significance for agricultural production to carry out research on soil salinization in coastal winter wheat planting areas and grasp the spatial distribution of salinization.

Traditional soil salt information is obtained mainly through field survey sampling and chemical analysis method. This method is relatively accurate, but it is time-consuming and labor-intensive, and has obvious limitations in terms of spatial globalization and effectiveness. In addition, field sampling can also cause damage to winter wheat and other crops. Satellite remote sensing data can make up for these shortcomings. Because of its high timeliness, economy and large-area simultaneous observation ability, it has become an important method for quantitative extraction of saline soil information in a large area. A large number of scholars have carried out related research and achieved excellent results. Most of them have realized a large-scale quantitative inversion of soil salt content based on multi-source satellite images [2,3,4,5,6,7,8,9,10,11], and they inverted soil salinity by studying the quantitative relationship between soil salinity and vegetation index. This is mainly because the vegetation cover and growth can reflect the status of soil salinity. In addition, the satellite image detects mainly the vegetation coverage information, so it is hard to directly use the spectrum of bare soil to monitor the soil salinity in the vegetation coverage area on a large scale [12,13,14,15]. When constructing soil salinity inversion model based on satellite imagery, most studies use traditional linear regression methods and machine learning algorithms. After comparison, it is found that most machine learning methods are more accurate and therefore more widely used [16,17,18,19].

However, satellite imagery is greatly affected by factors such as fixed orbit, time phase, weather, etc., especially the low spatial resolution and imaging quality, and it often fails to meet the demand of high-precision and real-time monitoring of soil salinization [4]. In recent years, UAV technology has rapidly developed, due to its advantages of high image accuracy, simple operation and flexibility, it has been applied to soil salt monitoring by scholars [20,21,22]. However, UAV technology has some limitations in monitoring soil salinization on a large scale due to its limited observation range. Therefore, it is necessary to make use of the complementarity of “Satellite-UAV-Ground” multi-scale data to carry out collaborative inversion of soil salinity.

At present, scholars have carried out certain researches on the inversion of surface-related parameters by combining data from two platforms such as Satellite-Ground and UAV-Ground. For example, An et al. [23] built soil salinity inversion model by simulating Landsat8 band through actual hyperspectral measurements on the ground. Schut et al. [24] combined the vegetable index of UAV and satellite with the crop growth model to evaluate the yield and fertilizer response in the field of heterogeneous smallholders, which would improve the accuracy of yield and crop production assessment. Kattenborn et al. [25] built a random forest model based on the spectrum, texture information and canopy structure obtained from UAV data, and upscaled it to Sentinel satellite data to draw a large range distribution map of tree species; Hu et al. [26] found that the inversion accuracy of soil salinization using the combination of UAV hyperspectral data and GF-2 multi-spectral data was better than that of GF-2 multi-spectral data inversion. Daryaei et al. [27] used Sentinel-2 and UAV data to conduct fine-scale monitoring of vegetation in semi-arid mountainous areas focusing on riparian landscapes, and the accuracy has been improved. Zhang et al. [2] established the reflectance relationship between satellite and UAV data and applied the constructed UAV high-precision model to satellite imagery to realize the SPAD worth inversion during the recovery period of winter wheat in a large area. Studies have shown that the combination of data from the two platforms can effectively improve the accuracy of the inversion, but most of these studies are based on the spectral information relationship between the data samples of different platforms for data fusion to achieve collaborative inversion. Due to the large spatial scale and spectral differences between the samples, it will cause the spectrum information loss and errors, which will affect the accuracy of inversion results. How to realize the accurate and efficient connection of data from the three platforms of “Satellite-UAV-Ground”, and then the high-precision and large-scale monitoring of ground surface information, especially soil salinization information, needs further research and exploration.

Therefore, this study selects typical coastal areas in the Yellow River Delta, and uses Sentinel-2, UAV and ground multi-platform data to explore its efficient and accurate collaborative method to carry out “Satellite-UAV-Ground” collaborative inversion of soil salinity in winter wheat planting areas. Thus, a rapid and accurate method for obtaining soil salt in the coastal area was proposed, which provided a scientific basis for the production and management of winter wheat.

## 2. Materials and Methods

### 2.1. Study Area

**Figure 1:**
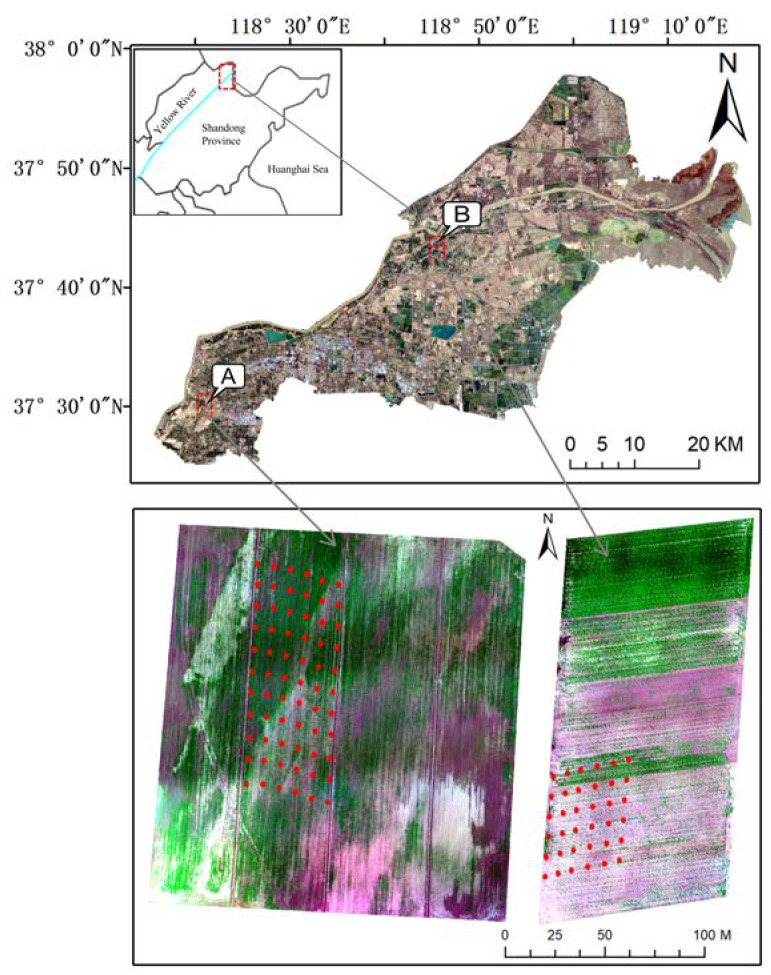
Location map of the study area and test areas (**A**, **B**), the red dots are the sampling points in the test area. The study area is true color image of Sentinel-2A, and the test area (**A**, **B**) are false color images of UAV.

The study area is located in Kenli District (37°24′–38°10′ N, 118°15′–119°19′ E), which is the core area of the Yellow River Delta. It has a warm temperate continental monsoon climate, with sufficient sunlight but little precipitation and evaporation large, uneven droughts and floods, and obvious seasonal alternating wet and dry. The annual mean precipitation, evaporation and air temperature are 511.6 mm, 1928.2 mm and 12.4 °C, respectively [28]. The terrain of the study area is low and flat, decreasing from southwest to northeast. The source of surface water is natural precipitation and water from the Yellow River. The groundwater level is relatively shallow and the salinity is high. The main soil type is gleyic solonchaks with a high sand proportion and high salinization. Soil texture is light, organic matter is generally lack, nitrogen and phosphorus are less, soil overall nutrient is poor, the pH value of the soil is greater than 7 [29]. The main crops are winter wheat, corn, rice and cotton, but the overall management is extensive and the yield is low. Wheat planting areas are mainly distributed in the higher terrain area in the southwest and the Yellow River coast area in the northeast. The variation of soil salinization is obvious, which is an ideal area for this study. Based on the investigation of Kenli area, landform, soil and crop distribution, two test areas A and B (Figure 1) were selected for the concentrated distribution of winter wheat in the southwest and northeast of the study area respectively to carry out field soil salinity measurement and UAV flight test. Test area A was a square area of 200 m × 200 m, and a 100 m × 50 m area was selected to arrange sampling points. Test area B was a rectangular area of 200 m × 100 m, and a 50 m × 50 m area was selected to arrange sampling points. An investigation suggests that planting time, farming methods, and fertilization conditions are all basically the same in A and B test areas, while the growth of winter wheat there is obviously different, with all soil salt content levels distributed, making the experimental area more typical and representative.

### 2.2. Data Acquisition and Preprocessing

#### 2.2.1. Acquisition of Ground Soil Salinity Data

There is less rainfall in spring in Kenli District, and the surface salinization is obvious and stable. Winter wheat is at the reviving stage, while other major crops are not sown, which is convenient for extracting spectral characteristics [30]. A field survey in the study area was conducted from April 10 to 16, 2019. To ensure uniform distribution, three samples were pre-arranged in the study area every 5 km × 5 km grid, and finally 77 winter wheat sample data were collected. At the same time, for the two test areas, a ground card was placed every 10m at the outer boundary of the sampling point area of the A and B test areas, and connect them with a measuring rope to form a 10 m × 10 m grid of sample points, taking the intersection of the grid as sampling points. This 10 m × 10 m grid is the same as the pixel size of Sentinel-2A. A total of 102 sample points was collected, including 66 in the A test area and 36 in the B test area. Four outliers in the sampling points were eliminated, and the remaining 98 samples were used to construct and verify the soil salinity inversion model of winter wheat. An EC110 portable salinity meter (Spectrum Technologies, Inc., Aurora, USA) equipped with a 2225FST series probe (conductivity temperature correction was performed) was used to make multiple measurements of the electrical conductivity (EC) of the soil surface layer 10 cm below the plant at each sample point and make a record after stabilization. The average of the measured values is taken as the EC value of each sample point, in ds/m. According to the results of earlier research which in our laboratory, the measured EC data were converted into soil salt content (SSC) in g/kg by using the formula SSC = 2.18 EC + 0.727 which was obtained by chemical analysis of soil in the same study area in spring [31]. At the same time, the orientation, topography, soil, wheat growth and other relevant information were recorded.

#### 2.2.2. Acquisition and Processing of UAV Imagery

A multispectral camera (Parrot Sequoia, Parrot Inc., Paris, France) was mounted on a Dajiang Matrice 600 Pro UAV (loaded mass: 5.5 kg; flying time: 18 min) (SZ DJI Technology Co., Ltd. Shenzhen, Guangdong Province, China). The camera can receive a total of 4 bands of information, which are green light (G), red light (R), red edge (RE), near infrared (NIR). The wavelengths are 550 nm, 660 nm, 735 nm, and 790 nm, and the band widths are 40 nm, 40 nm, 10 nm and 40 nm. The Sequoia multi-spectral camera is mounted on the head of the UAV, and the radiation sensor is mounted on the top of the UAV to write the radiometric correction data into the image during flight.

The data collection time was from 11:00 to 15:00 on 14 April 2019. The weather was clear and cloudless with low wind force when the UAV was flying. Before takeoff, the Sequoia multispectral camera and radiation sensor were calibrated, and the ground standard whiteboard image was collected. The flight height was 50 m, the flight speed was 5 m/s, and the image acquisition interval was 1.5 s. After the data are collected, they will be imported into Pix4D Mapper software (Pix4D, S.A., Prilly, Switzerland) for splicing, radiation correction and other processing to obtain the high-resolution orthophoto image of the test area, with a spatial resolution of 4–5 cm. Finally, in ENVI5.3, the decision tree method is used to remove the soil background. In order to eliminate the random error caused by the reflectance of a single point, a 5 × 5 pixels image is taken with the sampling point as the center, and the average reflectance value is taken as the reflectance data of the sampling point.

#### 2.2.3. Acquisition and Processing of Sentinel-2A Satellite Data

Sentinel-2 satellite is a multispectral imaging satellite with high resolution, revisit rate and update rate. It includes two small satellites A and B. The revisit period is 5 days. The main payload is MSI multispectral imager, covering 0.4–2.4 μm spectral range, including 10 m (four bands), 20 m (six bands), 60 m (three bands) ground resolution, which can monitor the growth, coverage and health of land vegetation, and obtain information on crop planting, land use changes, etc. In this paper, the Sentinel-2A products were downloaded from the ESA Copernicus data sharing website (https://scihub.copernicus.eu/). Considering the acquisition time of ground and UAV data and the quality of the image, the Sentinel-2A Level-1C multispectral image on 17 April 2019 was selected for modeling and inversion of the soil salt content, and the images of 3 November 2018 and 26 June 2019 were used for extraction of winter wheat planting areas.

The downloaded L1C data are orthophoto with geometric precision correction, without radiometric calibration and atmospheric correction. First, radiometric calibration and atmospheric correction are carried out by using Sen2cor, a plug-in published by ESA. Then, the data are re-sampled by the Sentinel Application Platform (SNAP) software to generate 10m spatial resolution images, and the data are exported in ENVI format. Finally, the Sentinel-2A true color images of the research area were obtained by splicing the images in ENVI 5.3 (Exelis Visual Information Solutions, Inc., Colorado, USA) and clipping the images using kenli District administrative boundary vector documents (Figure 1). In order to be consistent with the UAV image band, the green (G), red (R), red edge (RE), and near infrared (NIR) of Sentinel-2A image are selected in this study. The wavelengths are 560 nm, 665 nm, 740 nm, 865 nm, and the band widths are 45 nm, 38 nm, 18 nm and 33 nm.

### 2.3. Methods

#### 2.3.1. Calculation and Optimization of Vegetation Indices

Studies have shown that different levels of soil salinization have an impact on vegetation growth and morphology, plasma membrane permeability, photosynthetic pigments of leaves, gas exchange parameters, chlorophyll fluorescence characteristics, etc. [32]. Therefore, there are differences in spectral information of vegetation at different levels of soil salinization, which can indirectly reflect the level of soil salinization [33,34]. The vegetation index can highlight the characteristics of vegetation and effectively reflect the health and growth of vegetation. When the soil salt content increases, the reflectivity of visible red light of the salt-sensitive vegetation will increase, and the near-infrared reflectance will decrease [35]. In order to better reflect the vegetation conditions, 8 vegetation indexes related to red light and near-infrared are selected in this study, including normalized difference vegetation index (NDVI), normalized difference red edge index (NDRE), optimized soil adjusted vegetation index (OSAVI), green normalized difference vegetation index (GNDVI), triangle vegetation index (TVI), difference vegetation index (DVI), Improved chlorophyll absorption vegetation index based on PROSPECT and SAILH radiation transfer model (MCARI2) and renormalized difference vegetation index (RDVI).

The multi-band spectrum collected by the UAV is used to calculate the 8 vegetation indexes, and the formulas are shown in Table 1. Then the correlation coefficient between each vegetation index and soil salinity was calculated, and the variance inflation factor (VIF) between vegetation indexes was calculated by using the formula VIF = 1/(1-r*r) (r is the correlation coefficient between vegetation indexes) [12], excluding the low correlation or VIF > 10, which is the parameter that cannot be diagnosed by collinearity. The sensitive vegetation indexes are selected for soil salt modeling.

#### 2.3.2. Construction and Optimization of UAV-Ground Collaborative Inversion Model of Soil Salinity in Wheat Field Based on UAV Images

The 98 samples were sorted from small to large, and the modeling set and the validation set were sampled at equal intervals in a ratio of 2:1 to ensure the same range and uniform distribution of the model samples and the validation samples. 68 samples were selected for modeling and 30 samples for validation.

Taking the sensitive vegetation index as the input variable of the model, three methods were used to construct the winter wheat soil salinity inversion model, namely, BPNN, SVM and RF. BPNN is a multi-layer feedforward neural network trained according to the error back propagation algorithm, and it has also been applied to the salt inversion problem [39,40]. SVM is a new machine learning method from linear separable to linear nonseparable based on the principle of minimizing structural risk according to the statistical theory. It has been widely applied in image recognition and classification and has also been applied in regression problems in recent years [41,42]. The RF algorithm is an integrated learning algorithm obtained by combining the bagging algorithm with the decision tree algorithm. In recent years, many scholars have applied it to remote sensing technology [43,44]. All the three methods were implemented in MATLAB R2016b (MathWorks, Inc., Natick, MA, USA). The BPNN sets the number of training iterations to 1000, the accuracy to 0.003, the learning rate to 0.01; The SVM method was set as V-SVR, Gaussian kernel function was selected, the best penalty factor C and kernel parameter gamma were selected through network search and cross validation, and the SVM model was trained. The RF method called MATLAB random forest toolbox, and the parameter leaf node Leaf = 5 and the number of trees Ntrees = 200 were finally determined by Bayesian optimization.

The accuracy of model modeling and verification was evaluated by the coefficient of determination (R^2^) and root mean square error (RMSE) [45,46,47]. R^2^ is used to measure the fitting degree of the model, and RMSE reflects the deviation between measured value and predicted value. The closer R^2^ is to 1, the smaller the RMSE, which means the higher the accuracy of the model, the better the effect. The model with the best accuracy and effect was selected for soil salinity inversion of winter wheat. The degree of soil salinization is divided into five grades according to relevant criteria [48], non-salinization (<1 g/kg), mild salinization (1–2 g/kg), and moderate salinization (2–4 g/kg), severe salinization (4–6 g/kg) and saline soil (>6 g/kg), and we get the distribution map of soil salinity grade.

#### 2.3.3. Information Extraction of Winter Wheat Planting Area

**Figure 2:**
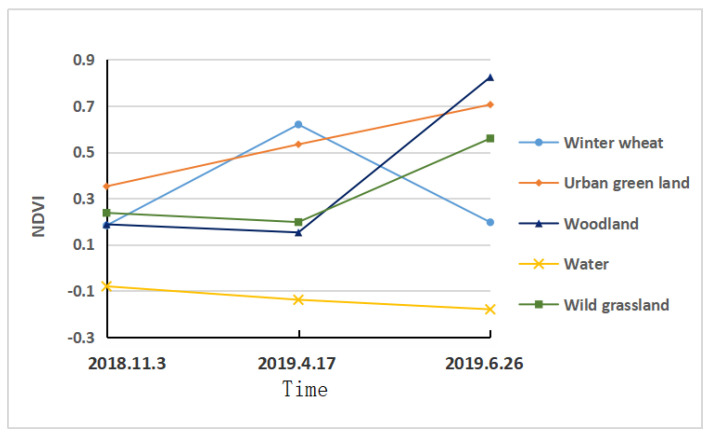
Changes of NDVI of different vegetation types in the study area with time.

The planting area of winter wheat in the study area was extracted by using the time series features composed of the NDVI of the Sentinel-2A images on 3 November 2018, 17 April 2019 and 26 June 2019. According to the investigation and analysis of various vegetation types in the study area, only winter wheat was in the seedling stage in early November, its growth reached the peak stage in late April of the second year, and entered the maturity stage at the end of June. Its NDVI time series curve showed a rapid rise from early November to late April, while other vegetation types showed little change in NDVI, the NDVI of winter wheat declined rapidly from late April to the end of June, while the NDVI time series curves of other vegetations were in varying degrees of rising stages, as shown in Figure 2. The valley-peak-valley in the NDVI time series at the beginning of November, late April and the end of June is a significant feature that distinguishes winter wheat from other vegetation. Therefore, by calculating the changes in NDVI from early November to late April, and late April to the end of June, a decision tree is established to extract this feature. Combined with the training sample data, the following decision rules are established:

(1) $$\mathrm{Types}\;\mathrm{of}\;\mathrm{ground}\;\mathrm{objects}=\left\{ \begin{array}{l} \mathrm{Winter}\;\mathrm{wheat},\;b_{2}>0.1\;and\;b_{2}-b_{1}>0.1\;and\;b_{3}-b_{2}<0\\ \mathrm{Other}\;\mathrm{ground}\;\mathrm{objects} \end{array}\right.$$

where *b*_1_, *b*_2_, and *b*_3_ are the NDVI values of Sentinel-2A images on 3 November 2018, 17 April 2019, and 26 June 2019, respectively.

Thus, the distribution of winter wheat planting areas on 17 April 2019 was obtained.

#### 2.3.4. Construction of the Satellite-UAV-Ground Collaborative Inversion Model of Soil Salinity in Wheat Area Based on Sentinel-2 Images

Sentinel-2A images have mixed pixels, and it is difficult to accurately obtain the measured data of soil salinity within the corresponding range of pixels. However, the resolution of UAV images is up to centimeter level, so the measured data of soil salinity within the corresponding range of pixels are easy to obtain and accurate. Therefore, the measured ground salt data and the UAV image vegetation index are used to construct a high-precision inversion model to obtain the soil salt content of the test areas A and B, then the salt values corresponding to sentinel-2 image pixels in the test area were calculated and taken as the “salt true value” of the Sentinel-2A image construction inversion model. It is combined with the Sentinel-2A image vegetation index to construct three inversion models including BPNN, SVM and RF.

**Figure 3:**
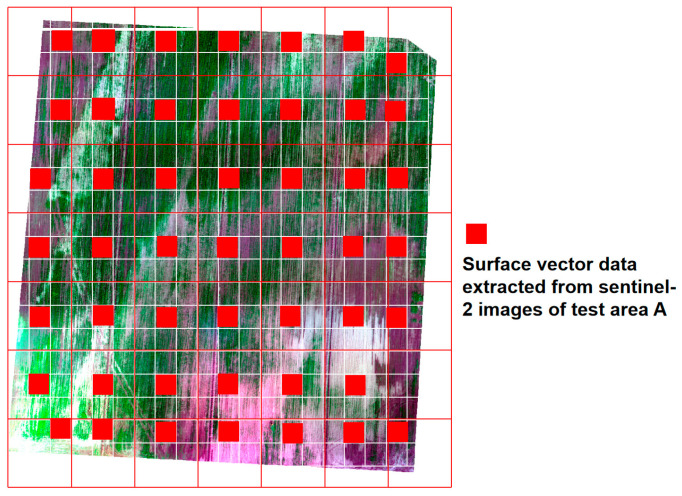
Distribution map of the extracted surface vector data of test area A.

First, the 10 m × 10 m surface vector data corresponding to the size positions of Sentinel-2A image pixels were successively established in the test area A and the test area B. In order to ensure the objectivity of the data, in the formed surface vector data grid, every three rows and three columns of surface vector data are used as a unit to extract the surface vector data at the center of the unit. If the center position is not in the unit, the surface vectors at other positions in the unit are extracted. Figure 3 is the distribution map of the area vector data extracted from the test area A and a total of 75 surface vector data are extracted. The vegetation index corresponding to the Sentinel-2A image pixel of the surface vector was counted and entered into the attribute information, and the combination of the vegetation indexes were used as the input variable of the model. Secondly, A vector surface corresponds to 40,000 image UAV pixels, and the average salt value of 40,000 UAV pixels is calculated as the salt “truth value” of the surface vector data. The soil salt content of 75 surface vector data was recorded into the attribute information as the output variable of the model. Finally, BPNN, SVM and RF were used to construct soil salt content collaborative inversion model based on Sentinel-2A image. All three methods were implemented in MATLAB R2016b.The BPNN sets the number of training iterations to 1000, the accuracy to 0.003, and the learning rate to 0.02; The SVM method was set as V-SVR, and the training set cross-validation and network search method were used to optimize the parameters. According to the principle of minimum variance, the penalty coefficient was determined as C = 10000, γ = 0.01; the RF method called MATLAB random forest toolbox, and the parameter leaf node, Leaf = 5, and the number of trees, Ntrees = 300, was finally determined by Bayesian optimization.

#### 2.3.5. Soil Salinity Inversion Results and Accuracy Analysis in Wheat Area

Among the three machine learning models constructed, the optimal model was selected according to R^2^ and RMSE. The optimal model was used to invert the soil salinity of the winter wheat planting area in Kenli District, and the soil salinity distribution map of winter wheat in Kenli District was obtained.

At the same time, this method was compared with the direct inversion method of satellite remote sensing, namely, based on Sentinel-2A images and ground-measured salt data, the optimal model was directly constructed and selected to invert the winter wheat soil salt distribution in Kenli District. In order to verify the accuracy of the two methods, the R^2^ and RMSE of the inversion results and the data of 77 winter wheat soil sampling points in Kenli District were calculated for quantitative evaluation.

## 3. Results and Analysis

### 3.1. Screening of Soil Salt-Sensitive Vegetation Index

Correlation analysis was conducted between UAV image vegetation index and measured soil salinity content respectively, as is shown in Table 2. In the correlation matrix, the DVI, MCARI2, and TVI of the eight vegetation indices had low correlations with soil salinity. The VIF value of OSAVI and NDVI was greater than 10, and there was strong multicollinearity. Therefore, the combination of vegetation index NDVI, NDRE, GNDVI and RDVI was selected as independent variables for modeling.

### 3.2. UAV-Ground Collaborative Inversion Model of Soil Salinity Based on UAV Images

Soil salinity inversion model was established by taking 4 vegetation indexes of 68 modeling samples as independent variables and soil salinity content as dependent variables, and 30 validation samples were used to verify the model. The results are shown in Table 3.

**Figure 4:**
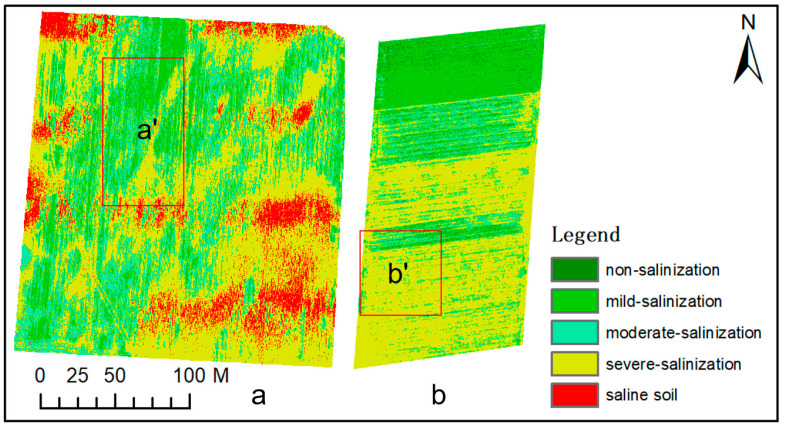

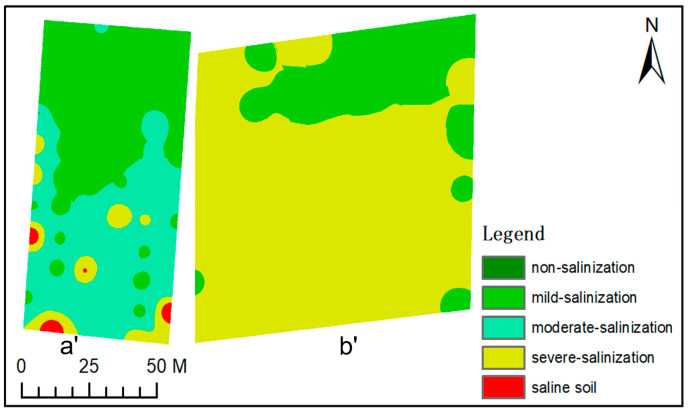
Salinity inversion map (**a**,**b**) and regional interpolation map (**a’**,**b’**) of sampling points in the test area.

It can be seen that all three inversion models show good stability, with their R_m_^2^ and R_v_^2^ both exceeding 0.6, and there is no “overfitting phenomenon”. The highest modeling R_m_^2^ of the RF algorithm is 0.878, which is 0.089 and 0.27 higher than the R_m_^2^ of the BPNN and SVM algorithms, and the RMSE_m_ is the lowest of 0.511, which is 0.159 and 0.38 lower than the RMSE_m_ of the BPNN and SVM algorithms, respectively. From the validation results, the highest R_v_^2^ of the RF algorithm is 0.827, which is 0.16 and 0.226 higher than the R_v_^2^ of the BPNN and SVM algorithms, and the RMSE_v_ is the lowest 0.473, which is 0.216 and 0.340 lower than the RMSE_v_ of the BPNN and SVM algorithms, respectively. The inversion model of the RF algorithm has the highest accuracy. Therefore, The RF inversion model was selected to conduct soil salinity inversion for UAV images in the test areas A and B, and the salinity levels were divided according to five levels to obtain the distribution diagram (Figure 4a,b). Figure 4a,b are the interpolation maps of the measured soil salinity in the sampling area. Generally speaking, the inversion results of the wheat field soil salinity are basically the same as the interpolation results of measured data, and the change trend of the area proportions of each grade is roughly the same, but in comparison, the reflection of the inversion results on spatial distribution of soil salinity is more refined. Therefore, the soil salinity inversion model of winter wheat based on RF is better and has the best stable effect.

### 3.3. Collaborative Inversion of Soil Salinity Based on “Satellite-UAV-Ground”

**Figure 5:**
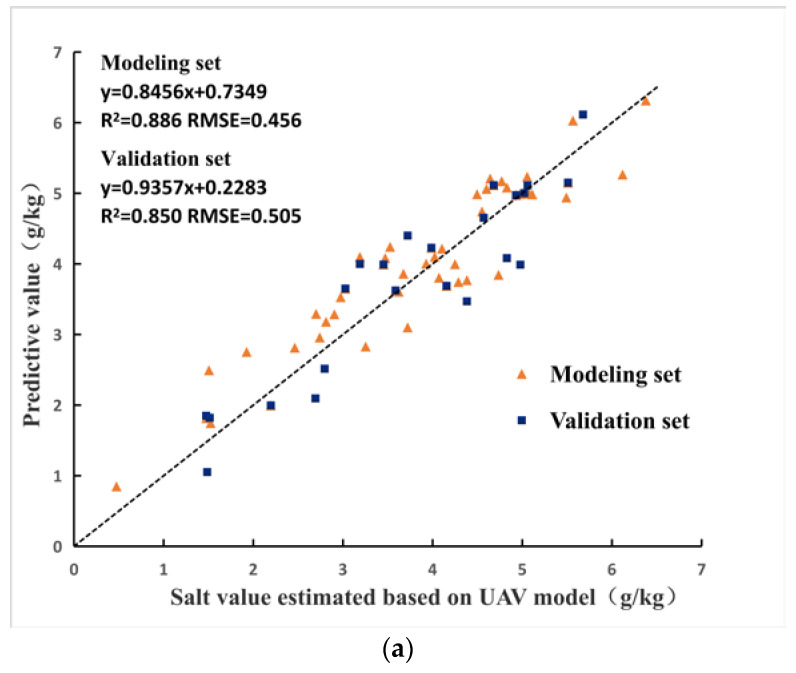

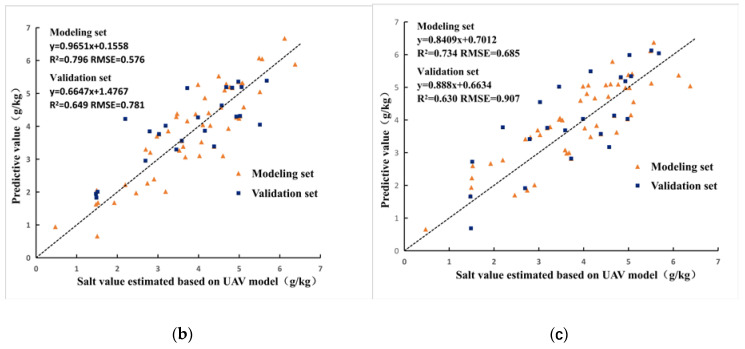
Accuracy of the three collaborative inversion models, (**a**) RF model, (**b**) SVM model, and (**c**) BPNN model.

Based on the 75 surface vector data of test areas, the NDVI, NDRE, GNDVI and RDVI of the Sentinel-2A images of 52 modeling samples were taken as independent variables, and the soil salt content estimated by UAV was taken as dependent variable to establish soil salt inversion model, and 23 validation samples were used to verify the model. Figure 5 shows the accuracy of the modeling set and validation set of the three models. By comparison, RF model has the highest accuracy, modeling set R^2^ = 0.886, RMSE = 0.456, validation set R^2^ = 0.850, RMSE = 0.505, followed by SVM model, the modeling set and validation set R^2^ are 0.796 and 0.649 respectively, and BPNN model has the lowest accuracy, the modeling set and validation set R^2^ are 0.734 and 0.630 respectively. Therefore, RF model is the optimal model for collaborative inversion of soil salinity based on “Satellite-UAV-Ground”.

#### 3.3.1. Analysis of Inversion Results of Soil Salinity in Wheat Area

**Figure 6:**
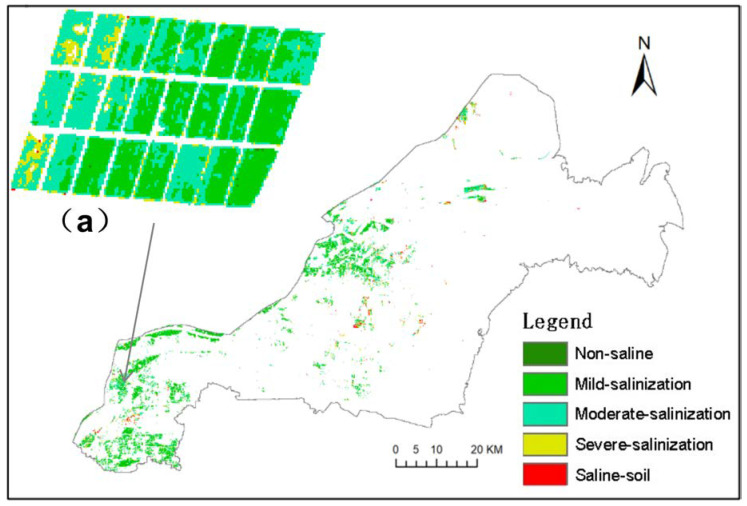
Inversion results based on “Satellite-UAV-Ground”, and (a) is a partial enlarged view.

A large-scale inversion of the soil salinity in the winter wheat planting area in Kenli district was carried out through the optimal RF model of the “Satellite-UAV-Ground” collaborative inversion, and the soil salinity level distribution map was obtained, as shown in Figure 6. Table 4 shows the area ratio of each salinity level. It can be seen that the distribution of non-saline soil in the wheat planting area of the study area is very small, accounting for only 0.05%. The proportion of winter wheat soil area shows a trend of decreasing with the increase of salinization, which is consistent with the actual situation of the survey. Among them, mildly salinized soil in wheat area is widely distributed, accounting for 61.32% of the total area. It is concentrated in the southwest where the terrain is relatively high and the northeast area affected by the fresh water of the Yellow River. The moderately salinized soil area was the second, accounting for 19.53%, which was distributed in all wheat regions. Severe salinized soil and salinized soil accounted for 19.1% of the total area and were scattered in wheat area.

#### 3.3.2. Accuracy Comparison between “Satellite-UAV-Ground” Collaborative and “Satellite-Ground” Direct Inversion

**Figure 7:**
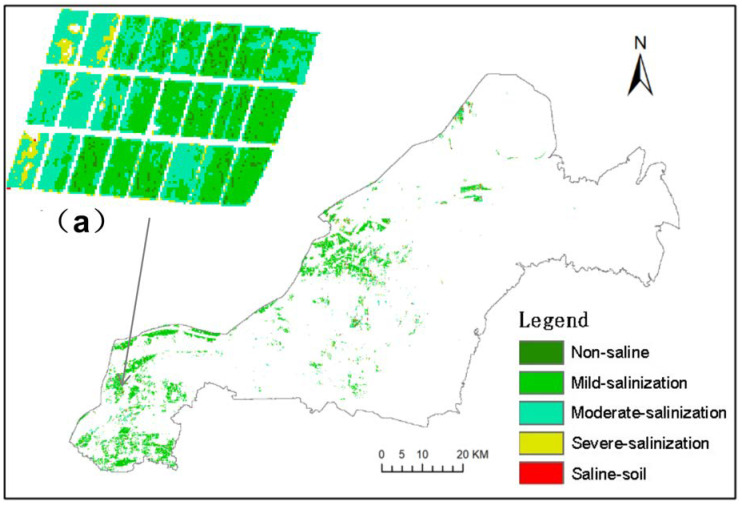
Inversion results based on satellite remote sensing, and (a) is a partial enlarged view.

Figure 7 shows the soil salinity inversion results of the RF model constructed directly based on Sentinel-2A images and measured salinity data. The R^2^ of the model is 0.56, which is far lower than the RF model of collaborative inversion. From the comparison of Figure 6 and Figure 7, it can be seen that the trend of soil salinity distributions obtained by the two inversion methods are basically consistent. However, Figure 7 is generally lower than the salt value in the survey. This is mainly due to the influence of the mixed pixels during the directly satellite remote sensing inversion method. Figure 6 has fewer non-saline areas and more saline soil areas, which is consistent with the field survey. As a result, using the UAV inversion result as the medium data can effectively reduce the influence of mixed pixels on the inversion result. 

**Figure 8:**
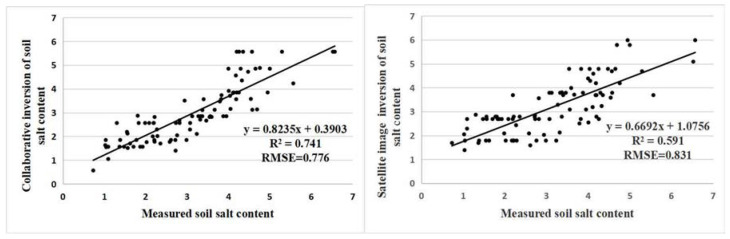
Scatter plots of measured sample points and soil salinity inversion results by two methods in Kenli District.

In order to further compare the inversion results of the “Satellite-UAV-Ground” collaborative method and the direct satellite remote sensing method, the salt values of 77 sampling points in Kenli District at the two inversion results were extracted and compared with the measured data. As is shown in Figure 8. The R^2^ = 0.741 and RMSE = 0.776 of the “Satellite-UAV-Ground” collaborative inversion results and the measured values, while the R^2^ = 0.591 and RMSE = 0.831 of the satellite remote sensing method and the measured values, indicating that the results of the “Satellite-UAV-Ground” collaborative inversion are highly consistent with the measured salinity, and the direct satellite remote sensing inversion results have a large deviation.

Therefore, “Satellite-UAV-Ground” collaborative inversion effectively improves the accuracy of soil salt inversion in winter wheat planting areas and obtains a more reliable soil salt distribution.

## 4. Discussion

The measured data on the ground is the basis for quantitative analysis of soil, UAV near-earth remote sensing is the link between the satellite and the ground, and satellite remote sensing is the platform for large regional inversion. Combining the three will be an important way to obtain soil salt information at present and in the future. Therefore, in this study, the three platforms of “Satellite-UAV-Ground” data were used to perform soil salinity inversion. Compared with the direct satellite image inversion results, the inversion quality was significantly improved. The inversion results of two models are verified with ground-based data. The results show that the R^2^ based on the “Satellite-UAV-Ground” collaborative inversion model has been significantly improved, and RMSE and distribution pattern is also improved, but not so obviously. It might be because the salinity inversion result of UAV image is used as an intermediate bridge, its scope is limited and it is hard to fully express the situation of the entire study area. We will continue to improve research on collaborative approach, models, vegetation spectra, data sampling, etc. to improve the performance in terms of scale, accuracy, and temporal and spatial resolution.

The selected test areas in this study were located in two concentrated winter wheat planting areas in the southwest and northeast of Kenli District. The growth of winter wheat was significantly different, and the soil salt content was distributed in all grades, which was typical and representative. In order to improve the spectral and salinity data accuracy of winter wheat, the grid intersection method was used to determine the location of ground data collection, which could ensure the uniformity and accuracy of sample points more than the traditional random sampling point determination method. In order to ensure the representativeness of modeling samples, the samples are sorted and sampled at equal intervals at the ratio of 2:1 between the calibration set and the validation set. Compared with the traditional method of random partition modeling set and validation set [5,49], the data is more uniform and effective, thus ensuring the strong universality and high stability of the model.

The soil salt contents in the study area vary greatly, so the smaller the pixel and the closer the sampling point, the more accurate the information reflected. In this study, an inversion model was built based on the measured ground data and UAV images. The salinity value obtained from the inversion was regarded as the “salinity truth value”, one pixel of Sentinel-2A corresponds to 40,000 UAV pixels, the average value of the soil salinity corresponding to these 40,000 UAV pixels was calculated, which was used as the salt value corresponding to the pixel of Sentinel-2A. Compared with the salt value measured on the ground, the “true salt value” is a more comprehensive representation of the true salt status in the Sentinel-2A pixel. Most of the previous studies have realized the up-scaling inversion method of UAV model by establishing the relationship of spectral information between UAV image and satellite image [5,50]. The method in this paper is a collaborative inversion model based on satellite images which is constructed by taking the UAV “salt truth value” as the bridge of “Satellite-UAV-Ground” collaborative inversion. It can better maintain the information content and spatial structure characteristics of the original remote sensing data, and better guarantee the accuracy of salt inversion. It is an effective way to realize the integrated inversion of “Satellite-UAV-Ground”.

Previous research results show that the machine learning inversion model is superior to the statistical model [51,52,53,54]. The machine learning method can show strong nonlinear fitting ability and excellent data mining ability and can better simulate the complex nonlinear relationship between soil salinity and remote sensing image characteristics. Therefore, three machine learning modeling methods were directly adopted in this study, and the comparison found that the RF model was particularly effective, especially the “Satellite-UAV-Ground” collaborative inversion model, with R^2^ and RMSE up to 0.886 and 0.456, respectively. Therefore, if there is a non-linear relationship between the predictor variable and the response variable, a non-linear model such as RF will usually have a better fitting effect and produce excellent estimation accuracy.

The growth of winter wheat in coastal salinized areas is mainly affected by soil salinity, while other factors such as soil texture, pH value, climate, water, nutrients, and fertility have a more balanced impact on crop growth, which is a systematic error. Therefore, salt content is mainly considered for the impact on vegetation index. The vegetation index can be used to invert soil salinity indirectly, which has been confirmed by previous research [11,55,56]. However, the relationship between the vegetation index and soil salinity is different under different environmental conditions. Consequently, the constructed model is only applicable to wheat fields in coastal saline soil in spring. In order to make the obtained soil salinity better support crop production, the next step is to perform salinity inversion for different crops in the study area in different seasons, and use satellite data at different times to verify the model to further improve the reliability of the model.

When salinity inversion is carried out by using data from different remote sensing platforms, matching between data is very important. The predecessors usually take the average of multiple measurements within the ground range corresponding to the satellite pixel as the salt value corresponding to the pixel [57,58]. In this paper, the salt value of the corresponding range of Sentinel-2A pixels is obtained by calculating the average value of 40,000 pixels of the corresponding UAV. Compared with the previous methods, the accuracy of salt value corresponding to satellite pixel is improved, thus the spatio-temporal matching accuracy of the data is improved. Due to the uncertainty of remote sensing data, the band response functions of different sensors are different. When building a collaborative inversion model, it is necessary to fully consider the radiation characteristics of the data and select similar bands to reduce the impact caused by radiation and improve the accuracy of data spectrum matching.

## 5. Conclusions

In this study, satellite images, UAV images and measured soil salt data were combined to build an inversion model to realize the “Satellite-UAV-Ground” collaborative inversion of soil salt in the coastal area of winter wheat. The main conclusions are as follows:

(1) The correlation between 8 vegetation indexes based on the multi-spectral bands of UAV images in the winter wheat test area and soil salinity was all greater than 0.5. According to the correlation coefficient and variance expansion factor, four sensitive vegetation indices, including NDVI, RDVI, GNDVI, and NDRE, were selected for modeling, and three machine learning inversion models, BPNN, SVM and RF were constructed. The RF model modeling set has R^2^ = 0.878, RMSE = 0.511, and its accuracy is higher than BPNN and SVM. It is the best salt estimation model. The inversion results are in good agreement with the actual distribution of soil salt in the test area. The model has good predictive ability and applicability for the estimation of soil salinity of winter wheat in spring in coastal salinization areas.
(2) The soil salinity in the test area obtained from the inversion of the best model of UAV imagery is used as the “true value of salinity” for satellite image modeling, and three collaborative inversion models are constructed. The RF inversion model has R^2^ = 0.885, which is significantly better than the BPNN and SVM models. The model is applied to the study area to obtain a large-scale distribution map of soil salinity in the winter wheat area. The soil in the winter wheat planting area of the study area is dominated by light and moderate salinization, accounting for 80.85% of the area, mainly distributed in the southwest and northeast regions. The area of heavily salinized and saline soil is smaller, only accounting for 19.1%, and is scattered in the wheat area.
(3) The result of “Satellite-UAV-Ground” collaborative inversion method and the satellite remote sensing direct inversion method were quantitatively compared and evaluated by using the measured salinity data of 77 sample points in the wheat field in the study area. The results show that the R^2^ = 0.741 and RMSE = 0.776 of the “Satellite-UAV-Ground” collaborative inversion result and the measured value, while the R^2^ = 0.591 and RMSE = 0.831 of the satellite remote sensing method and the measured value. Therefore, the “Satellite-UAV-Ground” collaborative inversion method can effectively improve the accuracy of the soil salt inversion results and obtain more accurate spatial distribution of winter wheat soil salt in spring in coastal salinization areas.

This study makes full use of the advantages of satellites, UAV images, and ground data to construct soil salinity inversion model, using UAV soil salinity inversion results as intermediate data, and machine learning modeling methods to obtain a grade distribution map of winter wheat soil salinity in the study area which is consistent with the actual distribution of soil salt. The study proposed an effective “Satellite-UAV-Ground” collaborative inversion method for soil salinity, which provides a scientific basis for accurately grasping the distribution of winter wheat soil salinity levels and guiding agricultural production in the study area.

## Figures and Tables

**Table 1:** Formulas and corresponding citation for vegetation indexes.

No.	Vegetation Index	Formula	Reference
1	NDVI	(NIR−R)/(NIR+R)	[36]
2	NDRE	(NIR−RE)/(NIR+RE)	
3	OSAVI	(1+0.16)(NIR−R)/(NIR+R+0.16)	
4	MCARI2	[3[(RE−R)−0.2(RE−G)(RE/R)]]/RE/R	
5	TVI	$\sqrt{\left(\mathrm{NIR}-R)/(\mathrm{NIR}+R\right)+0.5}$	[37]
6	DVI	NIR−R	
7	GNDVI	(NIR−G)/(NIR+G)	[38]
8	RDVI	$(\mathrm{NIR}-R)/\sqrt{\left(\mathrm{NIR}+R\right)}$	

**Table 2:** Correlation coefficient between vegetation index and soil salinity.

r	SS	NDVI	NDRE	GNDVI	OSAVI	RDVI	DVI	MCARI2	TVI
SS	1								
NDVI	−0.730 **	1							
NDRE	−0.669 **	0.709 **	1						
GNDVI	−0.710 **	0.928 **	0.691 **	1					
OSAVI	−0.699 **	0.961 **	0.666 **	0.931 **	1				
RDVI	−0.631 **	0.916 **	0.674 **	0.857 **	0.883 **	1			
DVI	−0.546 **	0.769 **	0.689 **	0.741 **	0.718 **	0.768 **	1		
MCARI2	−0.584 **	0.850 **	0.678 **	0.828 **	0.862 **	0.819 **	0.842 **	1	
TVI	−0.511 **	0.462 **	0.314 **	0.452 **	0.440 **	0.395 **	0.328 **	0.371 **	1

Significance levels: ** 0.01.

**Table 3:** Accuracy of three inversion models based on UAV images.

Modeling Methods	Modeling Set (*n* = 68)		Validation Set (*n* = 30)	
	R_m_^2^	RMSE_m_	R_v_^2^	RMSE_v_
BPNN	0.789	0.671	0.667	0.689
SVM	0.608	0.891	0.601	0.813
RF	0.878	0.511	0.827	0.473

**Table 4:** Statistics of soil salinity grade area in wheat area (Unit: %).

Soil Salinity Level	Non-Saline	Mild Salinization	Moderate Salinization	Severe Salinization	Saline Soil
Proportion of inversion result	0.05	61.32	19.53	12.28	6.82

## References

[1] Tian C.Y., Zhou H.F., Liu G.Q. (2000). Research suggestions on the regulation of soil salinization and sustainable agricultural development in Xinjiang in the 21st century. Arid Land Geogr..

[2] Zhang S., Zhao G., Lang K., Su B., Chen X., Xi X., Zhang H. (2019). Integrated Satellite, Unmanned Aerial Vehicle (UAV) and Ground Inversion of the SPAD of Winter Wheat in the Reviving Stage. Sensors.

[3] Allbed A., Kumar L., Aldakheel Y.Y. (2014). Assessing soil salinity using soil salinity and vegetation indices derived from IKONOS high-spatial resolution imageries: Applications in a date palm dominated region. Geoderma.

[4] Yao Y., Ding J.L., Zhang F. (2013). Regional soil salinization monitoring model based on hyperspectral index and electromagnetic induction technology. Spectrosc. Spect. Anal..

[5] Zhang S., Zhao G. (2019). A Harmonious Satellite-Unmanned Aerial Vehicle-Ground Measurement Inversion Method for Monitoring Salinity in Coastal Saline Soil. Remote Sens..

[6] Wang D.Y., Chen H.Y., Wang G.F., Cong J.Q., Wang X.F., Wei X.W. (2019). Research on UAV Multispectral Inversion of the Salt in the Severely Saline Soil of the Yellow River Estuary. Sci. Agric. Sin..

[7] Zhang T.-T., Qi J., Gao Y., Ouyang Z., Zeng S.-L., Zhao B. (2015). Detecting soil salinity with MODIS time series VI data. Ecol. Indic..

[8] Zhang T.R., Zhao G.X., Gao M.X., Chang C.Y., Wang Z.R. (2016). Salinity estimation and remote sensing inversion of winter wheat planting areas in the Yellow River Delta based on near-Earth multispectral and OLI images: Taking Kenli County and Wudi County in Shandong Province as examples. J. Nat. Resour..

[9] Chen H., Zhao G., Li Y., Wang D., Ma Y. (2019). Monitoring the seasonal dynamics of soil salinization in the Yellow River delta of China using Landsat data. Nat. Hazards Earth Syst. Sci..

[10] Abbas A., Khan S., Hussain N., Hanjra M.A., Akbar S. (2013). Characterizing soil salinity in irrigated agriculture using a remote sensing approach. Phys. Chem. Earth Parts A/B/C.

[11] Scudiero E., Skaggs T.H., Corwin D.L. (2015). Regional-scale soil salinity assessment using Landsat ETM + canopy reflectance. Remote Sens. Environ..

[12] Chen H.Y., Zhao G.X., Chen J.C., Wang R.Y., Gao M.X. (2015). Remote sensing inversion of saline soil salinity in the mouth of the Yellow River based on improved vegetation index. Trans. Chin. Soc. Agric. Eng..

[13] Yao Y., Ding J.L., Lei L. (2013). Monitoring spatial variability of soil salinity in dry and wet seasons in the North Tarim Basin using remote sensing and electromagnetic induction instruments. Acta Ecol. Sin..

[14] Zhang X.L., Zhang F., Zhang H.W., Li Z., Hai Q., Chen L.H. (2018). Optimization of hyperspectral index soil salinity inversion model based on spectral transformation. Trans. Chin. Soc. Agric. Eng..

[15] Guo P., Li H., Chen H.Y. (2018). Quantitative spectral estimation of soil salinity based on spectral index optimization. Bull. Soil Water Conserv..

[16] Wang F., Yang S.T., Ding J.L. (2018). Selection of environmentally sensitive variables and machine learning algorithm to predict oasis soil salinity. Trans. Chin. Soc. Agric. Eng..

[17] Jia P.P., Sun Y., Shang T.H., Zhang J.H. (2020). Estimation models of soil water-salt based on hyperspectral and Landsat-8 OLI image. J. Ecol..

[18] Wang M.K., Mo H.W., Chen H.Y. (2016). Study on the Modeling Method of Retrieving Soil Salinity Based on Multispectral Image. Soil Bull..

[19] Zhang S.M., Zhao G.X., Wang Z.R., Xiao Y., Lang K. (2018). Remote sensing inversion and dynamic monitoring of soil salinity in coastal saline area. J. Agric. Resour. Environ..

[20] Lin F., Zhao G.X., Chang C.Y., Wang Z.R., Li H. (2016). Area extraction and growth analysis of winter wheat based on adjacent orbit image. J. Agric. Resour. Environ..

[21] Schut A.G.T., Traore P.C.S., Blaes X., De By R.A. (2018). Assessing yield and fertilizer response in heterogeneous smallholder fields with UAVs and satellites. Field Crops Res..

[22] Ivushkin K., Bartholomeus H., Bregt A.K., Pulatov A., Franceschini M.H.D., Kramer H., Van Loo E.N., Roman V.J., Finkers R. (2019). UAV based soil salinity assessment of cropland. Geoderma.

[23] An D., Zhao G., Chang C., Wang Z., Li P., Zhang T., Jia J. (2016). Hyperspectral field estimation and remote-sensing inversion of salt content in coastal saline soils of the Yellow River Delta. Int. J. Remote Sens..

[24] Said N., Henning B., Joachim H. (2015). Estimation of soil salinity using three quantitative methods based on visible and near-infrared reflectance spectroscopy: A case study from Egypt. Arab J Geosci..

[25] Kattenborn T., Lopatin J., Förster M., Braun A.C., Fassnacht F.E. (2019). UAV data as alternative to field sampling to map woody invasive species based on combined Sentinel-1 and Sentinel-2 data. Remote Sens. Environ..

[26] Hu J., Peng J., Zhou Y., Xu D., Zhao R., Jiang Q., Fu T., Wang F., Shi Z. (2019). Quantitative Estimation of Soil Salinity Using UAV-Borne Hyperspectral and Satellite Multispectral Images. Remote Sens..

[27] Ardalan D., Hormoz S., Clement A. (2020). Fine-scale detection of vegetation in semi-arid mountainous areas with focus on riparian landscapes using Sentinel-2 and UAV data. Comput. Electron. Agric..

[28] Zhao Q., Bai J., Gao Y., Zhao H., Zhang G., Cui B. (2020). Shifts in the soil bacterial community along a salinity gradient in the Yellow River Delta. Land Degrad. Dev..

[29] Wen Y., Guo B., Zang W., Ge D., Luo W., Zhao H. (2020). Desertification detection model in Naiman Banner based on the albedo-modified soil adjusted vegetation index feature space using the Landsat8 OLI images. Geomat. Nat. Hazards Risk.

[30] Revill A., Florence A., MacArthur A., Hoad S.P., Rees R.W., Williams M. (2019). The Value of Sentinel-2 Spectral Bands for the Assessment of Winter Wheat Growth and Development. Remote Sens..

[31] Wang Z.R., Zhao G.X., Gao M.X., Chang C.Y., Jiang S.Q., Jia J.C., Li J. (2016). Spatial variability of soil water and salt in summer and soil salt microdomain characteristics in Kenli County, Yellow River Delta. Acta Ecol. Sin..

[32] Li J.L. (2013). Study on the Mechanism of the Influence of Salt in Coastal Saline Soil Cotton Field on Cotton Yield and Quality. Ph.D. Thesis.

[33] Jia J.C., Zhao G.X., Gao M.X., Wang Z.R., Chang C.Y., Jiang S.Q., Li J. (2015). Study on the relationship between winter wheat sowing area changes and soil salinity in typical area of the Yellow River Delta. J. Plant Nutr. Fertil..

[34] Zhang T.R., Zhao G.X., Gao M.X., Wang Z.R., Jia J.C., Li P., An D.Y. (2016). Estimation of soil salinity in typical areas of the Yellow River Delta based on near-surface multi-spectrum. Spectrosc. Spect. Anal..

[35] Tilley D.R., Ahmed M., Son J.H., Badrinarayanan H. (2007). Hyperspectral Reflectance Response of Freshwater Macrophytes to Salinity in a Brackish Subtropical Marsh. J. Environ. Qual..

[36] Dong C., Zhao G.X., Su B.W., Chen X.N., Zhang S.M. (2019). Research on the Decision Model of Variable Nitrogen Application in Winter Wheat’s Greening Period Based on UAV Multispectral Image. Spectrosc. Spect. Anal..

[37] Fang X.R., Gao J.F., Xie C.Q., Zhu F.L., Huang L.X., He Y. (2015). Survey of detection techniques and methods for crop canopy spectral information. Spectrosc. Spect. Anal..

[38] Dong J.J., Niu J.M., Zhang Q., KangSa R.L., Han F. (2013). Remote sensing yield estimation of typical grassland based on multi-source satellite data. Chin. J. Grassl..

[39] Yao R.J., Yang J.S., Zou P., Liu G.M. (2009). BP neural network model for spatial distribution of regional soil water and salinity. Acta Pedol. Sin..

[40] Zheng Z., Zhang F., Chai X., Zhu Z., Ma F. (2009). SPATIAL ESTIMATION OF SOIL MOISTURE AND SALINITY WITH NEURAL KRIGING. Stochastic Differential Systems.

[41] Diao S., Liu C., Zhang T. (2016). Extraction of remote sensing information for lake salinity level based on SVM: A case from Badain Jaran desert in Inner Mongolia. Remote Sens. Land Resour..

[42] Liang D., Guan Q.S., Huang W.J. (2013). Remote sensing inversion of leaf area index based on support vector machine regression in winter wheat. Trans. CSAE.

[43] Song R.J., Ning J.F., Chang Q.R. (2018). Kiwifruit orchard mapping based on wavelet textures and random forest. Trans. Chin. Soc. Agric. Mach..

[44] Yuan H., Yang G., Li C., Wang Y., Liu J., Yu H., Feng H., Xu B., Zhao X., Yang X. (2017). Retrieving Soybean Leaf Area Index from Unmanned Aerial Vehicle Hyperspectral Remote Sensing: Analysis of RF, ANN, and SVM Regression Models. Remote Sens..

[45] Zhou X.H., Zhang F., Zhang H.W. (2018). A Study of Soil Salinity Inversion Based on Multispectral Remote Sensing Index in Ebinur Lake Wetland Nature Reserve. Spectrosc. Spect. Anal..

[46] Xu K., Su Y., Liu J., Hu T., Jin S., Ma Q., Zhai Q., Wang R., Zhang J., Li Y. (2020). Estimation of degraded grassland aboveground biomass using machine learning methods from terrestrial laser scanning data. Ecol. Indic..

[47] Xie Y., Sha Z., Yu M., Bai Y., Zhang L. (2009). A comparison of two models with Landsat data for estimating above ground grassland biomass in Inner Mongolia, China. Ecol. Model..

[48] Bao S.D. (2000). Soil Agrochemical Analysis.

[49] Xi X., Zhao G.X. (2020). Retrieval and Monitoring of Chlorophyll Content in Winter Wheat Based on UAV Multi-spectral Remote Sensing. Chin. Agric. Sci. Bull..

[50] Chen J.Y., Wang X.T., Zhang Z.T., Han J., Yao Z.H., Wei G.F. (2019). Soil salinization monitoring method based on UAV-satellite remote sensing upscaling. Trans. Chin. Soc. Agric. Mach..

[51] Zhang Z.T., Wei G.F., Yao Z.H., Tan C.X., Wang X.T., Han J. (2019). Research on Soil Salt Inversion Model Based on UAV Multispectral Remote Sensing. Trans. Chin. Soc. Agric. Mach..

[52] Li Y.L., Zhao G.X., Chang C.Y., Wang Z.R., Wang L., Zheng J.R. (2017). Soil salt inversion model based on the fusion of OLI and HSI images. Trans. Chin. Soc. Agric. Eng..

[53] Yao Z.H., Chen J.Y., Zhang Z.T., Tan C.Y., Wei G.F., Wang X.T. (2019). Effect of plastic film mulch ing on soil salinity inversion by using UAV multispectral remote sensing. Trans. Chin. Soc. Agric. Eng..

[54] Qiu Y.L., Chen C., Han J., Wang X.T., Wei S.Y., Zhang Z.T. (2019). Satellite Remote Sensing Estimation Model of Soil Salinity in Jiefangzha Irrigation under Vegetation Coverage. Water Sav. Irrig..

[55] Wu W.C. (2014). The Generalized Difference Vegetation Index (GDVI) for Dryland Characterization. Remote Sens..

[56] Fernández-Buces N., Siebe C., Cram S., Palacio J.L. (2006). Mapping soil salinity using a combined spectral response index for bare soil and vegetation: A case study in the former lake Texcoco, Mexico. J. Arid Environ..

[57] Wang X., Zhang F., Ding J., Kung H.-T., Latif A., Johnson V.C. (2018). Estimation of soil salt content (SSC) in the Ebinur Lake Wetland National Nature Reserve (ELWNNR), Northwest China, based on a Bootstrap-BP neural network model and optimal spectral indices. Sci. Total Environ..

[58] Yue J., Yang G., Tian Q., Feng H., Xu K., Zhou C. (2019). Estimate of winter-wheat above-ground biomass based on UAV ultrahigh-ground-resolution image textures and vegetation indices. ISPRS J. Photogramm. Remote Sens..

